# A Case of Gitelman Syndrome with Hypercalcemia Secondary to Primary Hyperparathyroidism

**DOI:** 10.1155/2022/1098222

**Published:** 2022-06-25

**Authors:** Zeinab Alnahas, Marko Markov, Mohamad H. Horani

**Affiliations:** ^1^Department of Internal Medicine, Cairo University, Cairo 11956, Egypt; ^2^Chandler Regional Medical Center, Chandler 85224, Arizona, USA

## Abstract

Gitelman syndrome is a rare autosomal recessive salt-losing tubulopathy characterized by hypokalemia, hypomagnesemia, hypocalciuria, and secondary hyperaldosteronism. However, hypercalcemia secondary to hypocalciuria is extremely rare during the disease. A 36-year-old normotensive man who suffered a motor vehicle accident was presented with hypokalemia, hypomagnesemia, and mild hypercalcemia. He had a past medical history significant for bipolar depression disorder and a history of chronic atrial fibrillation. He was diagnosed with Gitelman syndrome. However, he was noncompliant with his medications. A laboratory workup revealed hypokalemia, hypomagnesemia, hypercalcemia, and a high parathyroid hormone level. Thorough investigations identified primary hyperparathyroidism as the primary cause of hypercalcemia. To our knowledge, Gitelman syndrome and primary hyperparathyroidism are an extremely rare association that is present in our case.

## 1. Background

Gitelman syndrome, also known as familial hypokalemia-hypomagnesemia, is a rare autosomal recessive hereditary disorder characterized by hypokalemic metabolic alkalosis, hypomagnesemia, hypocalciuria, and secondary hyperaldosteronism [1]. Gitelman syndrome tends to manifest during adolescence by various clinical presentations, including nonspecific symptoms such as fatigue, dizziness, nocturia, abdominal pain, muscle weakness, or muscle cramps and severe symptoms, such as tetany, paralysis, rhabdomyolysis, or fatal arrhythmia. However, individuals with Gitelman syndrome are usually asymptomatic and diagnosed by chance during routine blood testing [2]. The estimated prevalence of Gitelman syndrome is about 1 in 40,000 persons, with a significantly higher prevalence in the Asian population [3]. Although hypocalciuria is a distinctive characteristic of Gitelman syndrome caused by defective tubular reabsorption, it seldom can cause any change in the total plasma calcium level. Thus, the occurrence of hypercalcemia would require further investigation.

## 2. Case Presentation

We reported a 36-year-old normotensive man with a past medical history significant for bipolar depression disorder and gastroesophageal reflux disease and a history of atrial fibrillation. He was diagnosed with Gitelman syndrome based on the presence of hypokalemia, hypomagnesemia, and hypocalciuria. However, he was noncompliant with spironolactone, potassium, and magnesium supplementation. He suffered a motor vehicle accident, and on presentation to the emergency room, he was found with a serum potassium of 2.5 mmol/L (reference value 3.5–5.1 m·mol/L), serum magnesium of 0.8 mg/dL (reference value 1.7–2.2 mg/dL), total serum calcium of 11.3 mg/dL (reference value 8.4–10.2 mg/dL), serum phosphorus of 2.7 mg/dl (reference value 2.8–4.5 mg/dL), serum 25-dihydroxyvitamin D of 22.5 ng/mL (sufficient >25 ng/mL), random urine potassium of 122.1 mmol/L (reference value 20 mmol), 24-hour urinary calcium of 0.8 mg/kg/day (reference value < 4 mg/kg/day), and estimated fractional excretion of calcium of 0.004. He denied thiazide use, calcium, and vitamin D supplementation.

Further investigations were done to identify the potential causes of hypercalcemia and revealed a high intact parathyroid hormone of 144 pg/mL (reference value 10–65 pg/mL) consistent with primary hyperparathyroidism. His CT neck scan showed a 1.5 cm nodule inferior to the right thyroid lobe pole, consistent with an inferior parathyroid adenoma. However, a parathyroid radionuclide scan using technetium-99m MIBI showed no radiopharmaceutical localization for parathyroid adenoma within the neck or mediastinum. His abdominal CT scan excluded the presence of nephrolithiasis or nephrocalcinosis, and his echocardiography showed normal left ventricular function with no significant valvular pathology. Because the patient's serum calcium was higher than 1 mg/dL above the upper limits of the normal range, he underwent right inferior parathyroidectomy. The histological examination of the excised adenoma showed hypercellular parathyroid gland (678 milligrams) which confirmed the diagnosis of primary hyperparathyroidism. Shortly after the surgery, his serum calcium and parathyroid hormone levels were normalized, reaching 10 mg/dL and 56 pg/mL, respectively.

## 3. Discussion

Gitelman syndrome results from mutations at the SLC12A3 gene that encodes thiazide-sensitive sodium chloride cotransporter (NCCT) and magnesium channels in the renal distal convoluted tubule, causing salt-losing tubulopathy [4]. However, molecular genetic testing is not required for diagnosis, which can be established by the presence of the characteristic electrolyte imbalance [5]. Hypercalcemia is extremely rare in Gitelman syndrome, offset by the action of concomitant hypomagnesemia, which impairs the function of calciotropic hormones. This includes the function of parathyroid hormone (PTH) and 1,25-dihydroxyvitamin D [1,25 (OH)_2_D], resulting in decreased intestinal absorption of calcium. Lack of magnesium hinders the activation of 1 alpha-hydroxylase by PTH, leading to a decreased level of 1,25 (OH)_2_D. In addition, the negative relationship between circulating calcium levels and PTH is blunted in patients with Gitelman syndrome [6].

Therefore, the presence of hypercalcemia in patients with Gitelman syndrome requires further investigation to identify the potential causes of high calcium levels associated with elevated PTH levels, which may include primary hyperparathyroidism and familial hypocalciuric hypercalcemia. In primary hyperparathyroidism, there is a nonsuppressed secretion of PTH often from a single parathyroid adenoma, leading to high calcium levels. PTH stimulates osteoclast activity and suppresses osteoblast activity, resulting in bone breakdown and calcium release. Also, it increases renal calcium reabsorption and the formation of 1,25 (OH)_2_D, which stimulates intestinal calcium absorption [7]. The most common presentation of primary hyperparathyroidism is asymptomatic hypercalcemia. Nevertheless, it can cause a broad spectrum of clinical presentations, including fatigue, depression, memory loss, muscle and bone pain, and gastroesophageal reflux disease, or the classical manifestations of bones, stones, abdominal moans, and psychic groans [8]. In comparison, familial hypocalciuric hypercalcemia is a rare genetic disorder resulting from mutations in the calcium-sensing receptor (CaSR) gene and less commonly due to autoantibodies blocking CaSR. It is clinically similar to primary hyperparathyroidism, resulting in abnormal urinary calcium levels and high levels of serum calcium and PTH [9].

The presence of low urinary calcium excretion is the most important parameter to distinguish familial hypocalciuric hypercalcemia from primary hyperparathyroidism. However, in a patient with Gitelman syndrome, the differentiation between the possible causes of hypercalcemia based on the urine excretion of calcium is not reasonable due to the presence of underlying hypocalcuria caused by enhanced renal tubular absorption of calcium [10]. In such cases, family history and genetic testing would be helpful in the differentiation. Also, renal imaging and measurement of bone mineral density using a DXA scan are interesting for evaluating primary hyperparathyroidism complications and are normal in familial hypocalciuric hypercalcemia [11]. In our case, the presence of a high level of PTH and low-normal phosphorus in conjugation with the imaging findings of inferior parathyroid adenoma on the CT neck scan supported the diagnosis of primary hyperparathyroidism which has been confirmed by the histological examination and the normalization of serum calcium level after the surgery. Moreover, our patient's gastrointestinal, psychological, and cardiac symptoms could be related to hypercalcemia and hyperparathyroidism, either directly or through activation of neurotransmitters and sodium/potassium pump [12–14]. Although primary hyperparathyroidism is diagnosed based on biochemical findings, imaging studies are used for preoperative localization and are not diagnostic tools. A radionuclide technetium-99m MIBI scan could localize approximately 75% of solitary parathyroid adenoma [15].

## 4. Conclusion

Hypercalcemia is extremely rare in Gitelman syndrome, offset by the action of concomitant hypomagnesemia. Our case shows the importance of investigating the potential causes of hypercalcemia, such as hyperparathyroidism, in individuals with Gitelman syndrome. To our knowledge, Gitelman syndrome and primary hyperparathyroidism are an extremely rare association that is present in our case.
